# 2′-*O*-Methyl-8-methylguanosine as a Z-Form RNA Stabilizer for Structural and Functional Study of Z-RNA

**DOI:** 10.3390/molecules23102572

**Published:** 2018-10-09

**Authors:** Thananjeyan Balasubramaniyam, Takumi Ishizuka, Chao-Da Xiao, Hong-Liang Bao, Yan Xu

**Affiliations:** 1 Division of Chemistry, Department of Medical Sciences, University of Miyazaki, 5200 Kihara, Kiyotake, Miyazaki 889-1692, Japan; balasubramaniyam_thananjeyan@med.miyazaki-u.ac.jp (T.B.); tishizuka@med.miyazaki-u.ac.jp (T.I.); choutatsu_shou@med.miyazaki-u.ac.jp (C.-D.X.); xiaobujiao@163.com (H.-L.B.); 2 The key Laboratory of Optimal Utilization of Natural Medicine Resources, School of Pharmaceutical Sciences, Guizhou Medical University, University Town, Guian New District, Guiyang 550025, China

**Keywords:** Z-RNA structure, synthesis of oligonucleotide, NMR, circular dichroism

## Abstract

In contrast to Z-DNA that was stabilized and well-studied for its structure by chemical approaches, the stabilization and structural study of Z-RNA remains a challenge. In this study, we developed a Z-form RNA stabilizer m^8^Gm, and demonstrated that incorporation of m^8^Gm into RNA can markedly stabilize the Z-RNA at low salt conditions. Using the m^8^Gm-contained Z-RNA, we determined the structure of Z-RNA and investigated the interaction of protein and Z-RNA.

## 1. Introduction

The RNA helixes mostly adopt a right-handed A-from geometry. Another RNA conformation is characteristic of left-handed RNA having alternating CG base pairs [1,2]. Zα, a Z-form-binding domain of RNA-editing enzyme ADAR1, has been found to specifically bind to Z-DNA and Z-RNA [3,4,5,6,7,8,9]. It has been reported that Z-RNA participates in the interferon-response pathway [10,11] or viral inhibition [10,11,12]. The physical, chemical and spectral data on Z-RNA have been collected, whereas the biological role of Z-RNA has long been in question due to the difficulty of obtaining stable Z-RNAs under physiological salt conditions. For example, an A- to Z-RNA transition requires a very high salt concentration, e.g., 6 M NaClO_4_ [1,2].

Several studies have been reported on incorporating modified nucleic acid residues for the stabilization of Z-DNA [13,14,15,16,17,18,19,20,21]. Notably modified guanine residues highly favor the formation of Z-DNA. We have previously reported that the methylation of the C8 position of guanine significantly stabilized the Z-DNA because the modification by methylation was favorable for the *syn* conformation of the nucleobases [17]. Based on the results of these past studies, we have now developed a Z-form RNA stabilizer that stabilizes Z-RNA under physiological salt conditions. We designed and synthesized a 2′-*O*-methyl-8-methyl guanosine (m^8^Gm) by insertion of a methyl group at the C8 position of 2′-*O*-methyl guanosine. We found that incorporation of the m^8^Gm in the RNA dramatically stabilized the Z-RNA, even under physiological salt concentrations, and facilitated the A- to Z-RNA transition, even for AU-containing sequences that do not favor the formation of Z-RNA. We then determined the solution structure of r(CGC[m^8^Gm]CG)_2_. This allowed us to see the effect of the incorporation of a m^8^Gm on RNA and allowed us to understand the Z-RNA structure. The Z-RNA stabilizer can also be used to investigate the interaction of the Zα domain and Z-RNA.

## 2. Results and Discussion

We synthesized the m^8^Gm-incorporating oligonucleotides by phosphoramidite chemistry, using 2′-*O*-methyl guanosine (Schemes S1, S2 and Figures S1–S7). The induction of a methyl group in the C8 position highly favors the *syn* conformation. These structural features can lead the m^8^Gm to greatly stabilize Z-RNA. Using nuclear Overhauser effect (NOE) between C1′H and 8CH_3,_ we confirmed the *syn* conformation of m^8^Gm (Figure S8).

Circular dichroism (CD) spectroscopy has been used to study the Z-form conformation [17,22]. A-RNA, a negative Cotton effect appears around 295 nm, whereas in Z-RNA, a more positive band appears at 280 nm [23,24]. Thus, we performed CD spectroscopy experiments to study the conformation using various NaClO_4_ concentrations.

**Figure 1:**
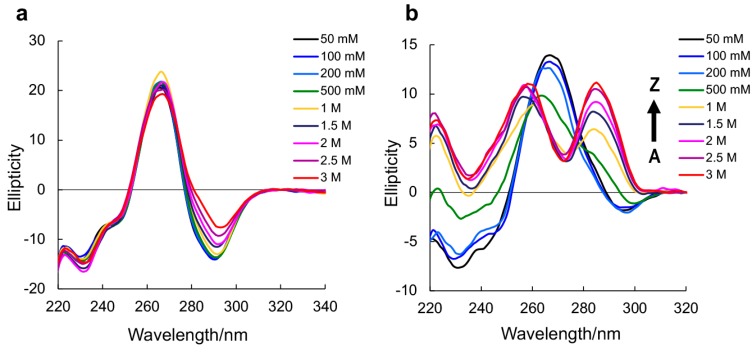
CD spectra of native RNA r(CGCGCG)_2_ (**a**) and m^8^Gm-contained r(CGC[m^8^Gm]CG)_2_ (**b**) (0.15 mM base concentration) at 10 °C with various NaClO_4_ concentrations in 5 mM sodium phosphate buffer (pH 7.0).

We used the CD spectroscopy to examine the A-Z transition under various salt concentrations. Native RNA r(CGCGCG)_2_ in A-form does not transfer to the Z-form, even in the presence of 3 M NaClO_4_ (Figure 1a). m^8^Gm-incorporated r(CGC[m^8^Gm]CG)_2_ showed a Z-form CD spectrum with increasing concentrations of NaClO_4_. (Figure 1b). The midpoint NaClO_4_ concentration for r(CGC[m^8^Gm]CG)_2_ was 880 mM, lower than that of the native RNA (4090 mM) (Table 1). r(C[m^8^Gm]C[m^8^Gm]CG)_2_ containing two m^8^Gms greatly stabilized the Z-form, showing a Z-form CD spectrum even in the presence of 50 mM NaClO_4_ (Figure S9) at a lower physiological salt concentration, and the midpoint was 100 mM (Table 1).

The incorporation of m^8^Gm can stabilize the Z-form containing an AU base pair. It showed the typical Z-form with 2360 mM midpoint compared to the native RNA (Figure S9 and Table 1). Octamer RNA containing four m^8^Gms in double strands also effectively stabilized the Z-form with 850 mM midpoint.

We designed a short duplex containing only one modified base, r(C[m^8^Gm]CGU[m^8^Gm]CG)/r(CGCACG), in which the underlined sequences can form a duplex containing only one m^8^Gm base. We compared its stability with native RNA r(CGCGUGCG)/r(CGCACG). Although the 6 base pairs duplex containing an AU base pair does not favor the formation of Z-RNA, it facilitated the A- to Z-RNA transition as compared to native 6 base pairs RNA (Figure S10). The midpoint for RNA containing one m^8^Gm was 4000 mM, lower than that of the native RNA (>6000 mM) (Figure S10), indicating that only one m^8^Gm can stabilize the Z-form, indicating that only one m^8^Gm can stabilize the Z-form.

**Figure 2:**
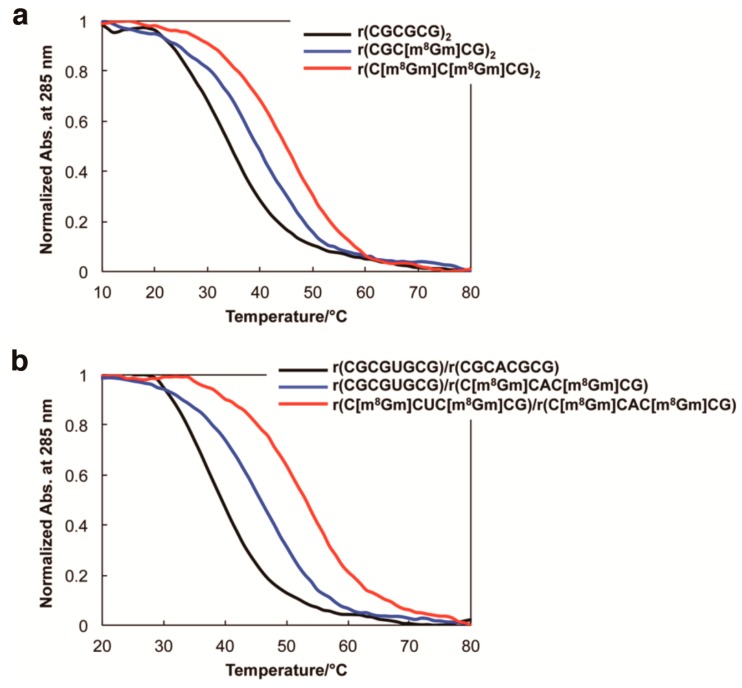
(**a**) CD melting curves for native RNA r(CGCGCG)_2_ (back line), r(CGC[m^8^Gm]CG)_2_ containing one m^8^Gm (blue line), and r(C[m^8^Gm]C[m^8^Gm]CG)_2_ containing two m^8^Gms (red line). (**b**) CD melting curves for RNA sequences containing an AU base pair, native RNA r(CGCGUGCG)/r(CGCACGCG) (back line), r(CGCGUGCG)/r(C[m^8^Gm]CAC[m^8^Gm]CG) having two m^8^Gms in one strand (blue line), r(C[m^8^Gm]CGU[m^8^Gm]CG)r(C[m^8^Gm]CAC[m^8^Gm]CG) having four m^8^Gms in two strands (red line). In 5 mM sodium phosphate buffer (pH 7.0), 7 M NaClO_4_ concentrations (0.15 mM base concentration).

To further understand the m^8^Gm effect on the thermodynamic properties of Z-RNA, the melting temperature (*T*_m_) and thermodynamic parameters were examined using CD melting experiments. (*T*_m_) values of m^8^Gm-incorporated hexamer RNA increased by 6–15 °C (Δ*T*_m_), (Figure 2a and Table 2), and octamer RNA with four m^8^Gms in double strands containing an AU base pair increased even more, by 15 °C (Figure 2b and Table 2). Thermodynamic parameters revealed a very favorable free energy of formation for the m^8^Gm-incorporated Z-RNA compared to the native RNA (Table 2) suggesting that the *syn* conformation of m^8^Gm was thermodynamically favorable, and the preferred C3′ *endo* conformation of ribose induced by the 2′-*O*-methyl group also contributed to the stabilization of the Z-form, which is consistent with the previous study [1,25]. The advantages of 2′-*O*-methyl substituent include provision of an NMR signal from 2′-*O*-methyl group for NMR structure study, easy-to-prepare phosphoramidite reagent more than 2′-*O*-hydroxy group for the chemical introduction of m^8^Gm into RNA oligonucleotides, and efficient resistance to enzymatic cleavage of oligonucleotide [1,26,27].

**Figure 3:**
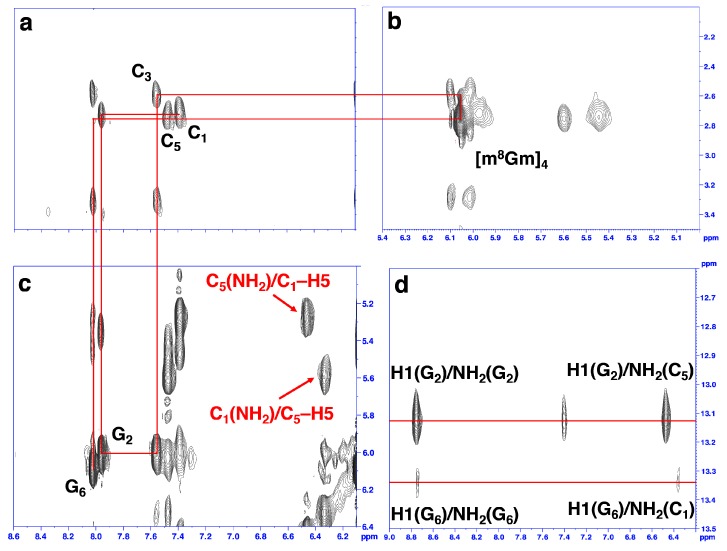
(**a**–**c**) H6/H5′′ of C and H8/H1′ of G (8CH_3_/H1′ of m^8^Gm) proton region of NOESY spectra of r(CGC[m^8^Gm]CG)_2_ in NaClO_4_ solution. The NOE connectivity pathway is shown as red line. Intraresidue NOE cross-peaks are labeled with residue numbers. The Z-RNA structure specific cross-peaks were observed between C_5_ amino protons and C_1_H5 protons from the opposite strand (indicated as red words), as well as between C_1_ amino protons and C_5_H5 protons from the opposite strand (**c**). (**d**) The cross peaks of imino proton of G_2_ and amino proton of C_5_, as well as imino proton of G_6_ and amino proton of C_1_, indicated Watson–Crick-type base pairs of Z-RNA. Intraresidue NOE cross-peaks of imino and amino proton of G_2_ and G_6_ are shown.

In order to obtain the structural information of Z-RNA, we performed an analysis of the 2D NOESY spectra of r(CGC[m^8^Gm]CG)_2_. A complete list of ^1^H chemical shifts was shown in Table S1. The NOE-restrained refinement provided an unequivocal demonstration that the structure of r(CGC[m^8^Gm]CG)_2_ is Z-RNA. We observed the strong cross-peaks in 2D-NOESY spectrum from intranucleotide H1′/H8 NOEs of G_2_ and G_6_ and a special NOE between C1′H and 8CH_3_ of m^8^Gm_4_. This indicated the *syn* conformation of all three guanosine residues (Figure 3b,c).

The NOE connectivity path between H6/H5′′ of C and H8/H1′ of G (8CH_3_/H1′ of m^8^Gm_4_) allows for a cross-peak assignment of Z-RNA nonexchangeable resonances (Figure 3a–c). Sequential assignments of C_1_ to G_6_ for the Z-form can complete the pathway: C_1_(H6/H5′′)-G_2_(H8/H1′)-C_3_(H6/H5′′)-m^8^Gm_4_(8CH_3_/H1′)-C_5_(H6/H5′′)-G_6_(H8/H1′) (Figure 3a–c), indicating a sequence-specific connectivity for left-handed helices.

The H′_2_ resonance was assigned based on NOESY spectra for sequential connections between H6/H8 and H′_2_ of C and G (Figure S11). Moreover, the clear cross-peaks of the imino proton of G (around 13.0 ppm) and the amino proton of C suggested WC base pairs (Figure 3d). The 2D NOESY of the imino protons and other protons was used for assignment. For example, we note that the C_5_(NH_2_) amino proton has cross-peaks (C_5_(NH_2_)/C_1_–H5) to the C_1_–H5 proton, and the C_1_(NH_2_) amino proton has cross-peaks to the C_5_–H5 proton (C_1_(NH_2_)/C_5_–H5) (Figure 3c). Such cross-peaks can only happen between the two interstrand cytosines between C1 in one strand and C5 in another strand of the Z-RNA because of their base pair stacking pattern [28,29].

**Figure 4:**
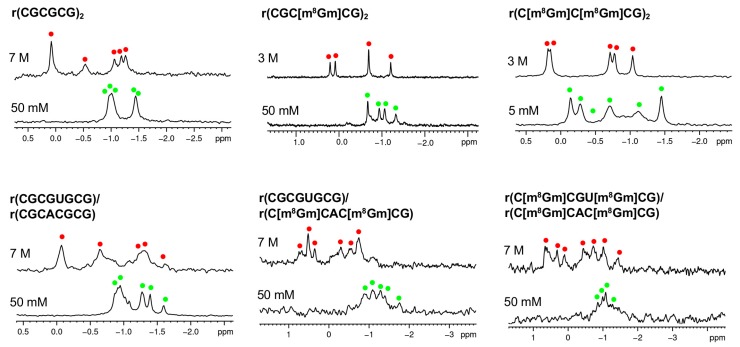
Monitoring of the A to Z-RNA transition by ^31^P NMR. ^31^P NMR spectra of the RNAs at 5 mM-7 M NaClO_4_. Green and red dots indicate the ^31^P peaks resulting from A-RNA and Z-RNA, respectively.

We further used ^31^P-NMR spectroscopy to confirm these observations. We found that these sequences as shown in Table 2 undergo a A-Z transition with increasing NaClO_4_ from 5 mM to 7 M (Figure 4). These results are consistent with the conformational data from the CD and proton NMR results.

**Figure 5:**
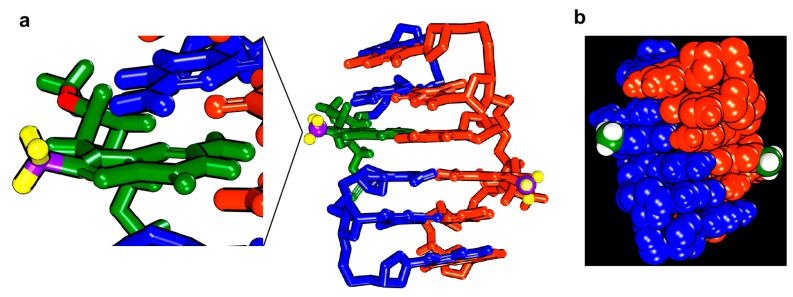
The model for r(CGC[m^8^Gm]CG)_2_ Z-RNA structure. (**a**) m^8^Gm is expanded in green with the C8-methyl group, hydrogen and carbon of methyl group are coloured in yellow and purple. (**b**) Hydrogen and carbon of methyl group are coloured in green and white. C8-methyl groups were located outside of Z-RNA.

A model of r(CGC[m^8^Gm]CG)_2_ was constructed based on the reported Z-form structure and NOE-constrained refinement [2]. The molecular dynamics simulations were performed by the standard dynamics cascade in BIOVIA Discovery Studio 4.5 with some modifications. The conformation with the lowest energy was selected as shown in Figure 5. In the m^8^Gm-contanied Z-RNA, the C8-methyl groups were hydrophobic and located in the outside of the helix, which is consistent with the induction of a methyl group that strongly contributes to the increased stabilization of Z-RNA.

**Figure 6:**
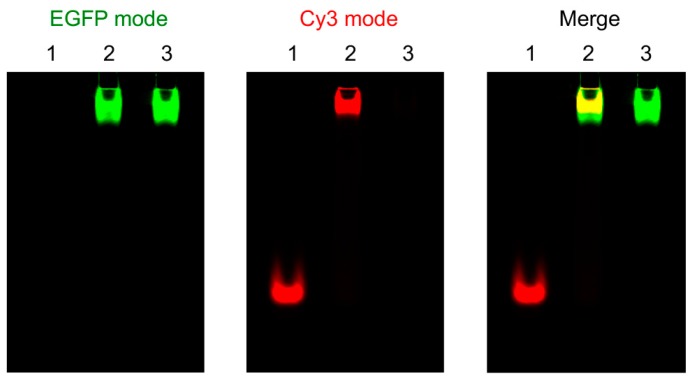
Visualization of Z-RNA and Zα-EGFP protein. Lane 1: Z-RNA only, Lane 2: RNA + Zα-EGFP, Lane 3: Zα-EGFP only. The different modes are indicated in upper. [RNA] = 1 μM, [Zα-EGFP] = 10 μM, [NaCl] = 100 mM, [Tris-HCl (pH7.0)] = 10 mM, [DTT] = 5 mM, 10% glycerol, 10 μg/mL BSA. Z-RNA: r(CGCGUGCG)-Cy3/r(C[m^8^Gm]CAC[m^8^Gm]CG).

Encouraged by the ability to use m^8^Gm to stabilize Z-RNA, we study the interaction of the Zα domain and Z-RNA by using a m^8^Gm-containing a Z-RNA and Zα-EGFP fusion protein, in which the Zα domain is tagged with a green fluorescence protein [29]. Figure 6 indicates the visualized mode as EGFP-mode, Cy3-mode and Merge, respectively. Lane 1, lane 2 and lane 3 in Figure 5 show free Z-RNA labeling with fluorescent dye Cy3, a complex of Z-RNA and Zα-EGFP and free Zα-EGFP, respectively. Green and red fluorescence emission visualized under EGFP-mode and Cy3-mode distinguished Zα-EGFP and Z-RNA. Because of the large molecular weight of Zα-EGFP, we visualized the complex of the Zα-EGFP and Z-RNA in the upper. Lane 2 in merge mode of Figure 6 emitted yellow fluorescence and clearly demonstrated the complex of Zα with Z-RNA. These observations indicated that Zα-EGFP efficiently binds to the m^8^Gm-containing Z-RNA without severe steric clashes with the added methyl groups, suggesting the m^8^Gm stabilized Z-RNA can be used to investigate the interaction of proteins with Z-RNA.

Recent studies suggested that dsRNA pathways of RNA silencing and micro-RNA regulation were interfered with by ADAR1 [29]. Zα is also reported to enhance ADAR1 editing activity on RNAs with Z-forming sequences in vitro [29]. This suggested that Z-RNA plays important biological roles similar to Z-DNA.

In conclusion, the results described here reveal that the newly synthesized guanosine analogue 2′-*O*-methyl-8-methyl guanosine dramatically stabilizes Z-RNA, which arises from the *syn* conformation of the m^8^Gm base and the C3′ *endo* conformation of ribose. The oligonucleotides with AU base pairs can convert into Z-RNA. In addition, researchers can utilize the Z-RNA stabilizer to study the interaction of Z-RNA sequences with the Zα domain. Using the Z-RNA stabilizer, we can refine the structure of Z-RNA based on NMR and examine the molecular basis of Z-form-specific interactions and reactions.

## 3. Materials and Methods 

### 3.1. Sample Preparation

We synthesized the RNA oligonucleotides by phosphoramidite chemistry with a DNA/RNA synthesizer. We purified by RP-HPLC and prepared Zα-EGFP fusion protein according to the method of Oyoshi et al. [15].

### 3.2. CD Experiments

We carried out CD experiments by a JASCO J-820 CD spectrophotometer (JASCO Corporation, Tokyo, Japan). The melting curves were obtained by monitoring 285 nm. 0.3 mL samples at 0.15 mM base concentration with 0.1–7 M NaClO_4_, 5 mM sodium phosphate buffer (pH 7.0).

### 3.3. NMR Experiments

We performed NMR experiments on a Bruker AV-400 M spectrometer (Bruker, Billerica, MA, USA). A special Micro Tube that is designed for use with reduced sample volumes was used (catalog no: NE-H5/3-Br, New Era NMR). Dissolve RNA samples (1.0 mM) in 150 μL of 90% H_2_O/10% D_2_O, 5 mM sodium phosphate buffer (pH 7.0), 50 mM or 3 M NaClO_4_. 2D NOE spectra were recorded with mixing times of 500 ms.

### 3.4. Molecular Modeling

The model of RNA structure was manually generated based on the reported structure using the BIOVIA Discovery Studio 4.5 (Accelrys, San Diego, CA, USA). Then molecular dynamics simulations were performed by the standard dynamics cascade in BIOVIA Discovery Studio 4.5 with some modifications. The structure was heating from 50 K to 300 K over 4 ps and equilibration at 300 K with 100 ps simulation time. The save results interval in the production step was 2 ps during 100 ps simulation time at 300 K. 10 best conformations generated by simulation were further energy minimized. The conformation with lowest energy was selected as shown in Figure 5.

### 3.5. Elctrophoretic Mobility Shift Assay (EMSA)

We performed the experiments using RNA containing m^8^Gm and Cy3-labeled RNA (1 μM each) with Zα-GFP fusion protein (10 μM) in a buffer, 10 μg/mL BSA and 10% (*v/v*) glycerol, 100 mM NaCl, 10 mM dithiothreitol, 10 mM Tris-HCl (pH 7.0). Nondenatured polyacrylamide gel (8%) is used and performed at 80 V and 4 °C in 1× TBE buffer with 20 mM NaCl.

## Figures and Tables

**Table 1:** Midpoint NaClO_4_ concentration in A-Z transition.

Oligonucleotides	NaClO_4_ (mM)
r(CGCGCG)_2_	4090
r(CGC[m^8^Gm]CG)_2_	880
r(C[m^8^Gm]C[m^8^Gm]CG)_2_	100
r(CGCGUGCG)/r(CGCACGCG)	5430
r(CGCGUGCG)/r(C[m^8^Gm]CAC[m^8^Gm]CG)	2360
r(C[m^8^Gm]CGU[m^8^Gm]CG)/r(C[m^8^Gm]CAC[m^8^Gm]CG)	850

**Table 2:** Thermodynamic parameters for A-Z transition of various m^8^Gm-containing RNAs.

Oligonucleotides	*T*_m_^1^ (°C)	Δ*T*_m_ (°C)	∆G_29__8__K_ (kcal/mol)
r(CGCGCG)_2_	33.5	−	−1.1
r(CGC[m^8^Gm]CG)_2_	40.0	+6.5	−1.7
r(C[m^8^Gm]C[m^8^Gm]CG)_2_	45.7	+12.2	−2.1
r(CGCGUGCG)/r(CGCACGCG)	37.7	−	−1.7
r(CGCGUGCG)/r(C[m^8^Gm]CAC[m^8^Gm]CG)	45.7	+8.0	−2.5
r(C[m^8^Gm]CGU[m^8^Gm]CG)/r(C[m^8^Gm]CAC[m^8^Gm]CG)	52.7	+15.0	−3.3

^1^ Measurement condition: [RNA] = 0.15 mM base concentration, [sodium phosphate buffer (pH 7.0)] = 5 mM, [NaClO_4_] = 7 M.

## Supplementary Materials

The following are available online. Schemes S1 and S2: Synthetic scheme of compound **5** and **6**, Figures S1–S6: ^1^H-NMR spectra of synthesized compounds, Figure S7: HRMS spectra of synthesized compounds, Figure S8: NOESY spectrum of m^8^Gm, Figure S9: CD spectra of RNA shown in Table 1 at various NaClO_4_ concentrations, Figure S10: H6/H2′ of C and H8/H2′ of G (8CH_3_/H2′ of m^8^Gm) proton region of NOESY spectra of r(CGC[m^8^Gm]CG)_2_, Figure S11: Monitoring of the A to Z-RNA transition by ^31^P-NMR, Table S1: ^1^H-NMR chemical shifts δ_H_ (p.p.m. of r(CGC[m^8^Gm]CG)_2_ Z-RNA.
