# Supplementary material for: 2′-O-Methyl-8-methylguanosine as a Z-Form RNA Stabilizer for Structural and Functional Study of Z-RNA
Source: Molecules. 2018 Oct 9;23(10):2572. doi: 10.3390/molecules23102572 (PMC6222775; doi:10.3390/molecules23102572)
Supplement: Supplementary file 1 [file molecules-23-02572-s001.pdf]

Supplementary Materials

2′-*O*-Methyl-8-methylguanosine as a Z-form RNA Stabilizer for Structural and Functional Study of Z-RNA

Thananjeyan Balasubramaniyam [1], Takumi Ishizuka [1], Chao-Da Xiao [1,2], Hong-Liang Bao [1], and Yan Xu [1,*]

1 Medical Sciences, Faculty of Medicine, University of Miyazaki, 5200 Kihara, Kiyo-take, Miyazaki 889-1692, Japan; balasubramaniyam_thananjeyan@med.miyazaki-u.ac.jp (T.B.); tishizuka@med.miyazaki-u.ac.jp (T.I.); choutatsu_shou@med.miyazaki-u.ac.jp (C.-D.X.)

2 The key laboratory of optimal Utilizaiton of Natural Medicine Resources, School of Pharmaceutical Sciences, Guizhou Medical University, University Town, Guian New District, Guizhou 550025, China; xiaobujiao@163.com

* Correspondence: xuyan@med.miyazaki-u.ac.jp; Tel.: +81-985-85-0993

General: DMSO-d_6 and $CDCl_3$ were used as the solvents. ^1H spectra chemical shifts (δ) are reported in parts per million (ppm) referenced to residual protonated solvent peak (DMSO-d_6, δ = 2.50, $CDCl_3$, δ = 7.26). Coupling constants (*J*) values are given in hertz (Hz). Signal patterns are indicated as br (broad), s (singlet), d (doublet), t (triplet), sept (septet), m (multiplet). ^1H-NMR and ^{31}P-NMR spectra were recorded on a BRUKER (AV-400M) magnetic resonance spectrometer. All reagents were purchased from Aldrich, TCI (Tokyo Chemical Industry) or Wako (Wako Pure Chemical Industries). Thin layer chromatography (TLC) was performed using TLC Silica gel 60 F_{254} (Merck). Compounds were visualized using a UV lamp (254 nm) or staining with a potassium permanganate solution. Silica gel (Wakogel® C-300, 200–325 mesh) was used for column chromatography. Purification of products was also performed on a middle pressure liquid chromatography (MPLC) systems (EPCLC-AI-580S, Yamazen Corporation) equipped with silica gel column (Hi-Flash Column, Yamazen Corporation). High-resolution mass spectra (HRMS) were recorded by electrospray ionization (ESI) on an Exactive Orbitrap mass spectrometer instrument (Thermo Scientific).

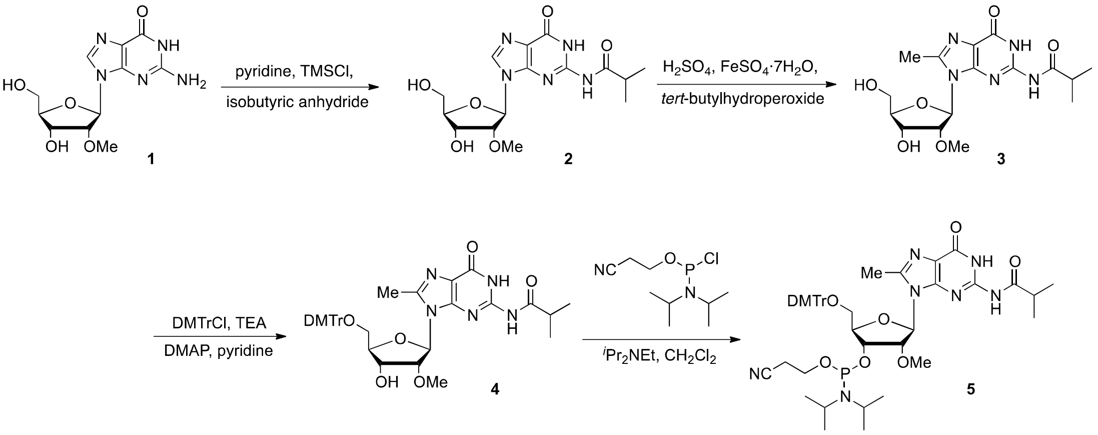

Scheme S1. Synthetic scheme of 2′-*O*-methyl-8-methylguanosine phosphoramidite **5.**

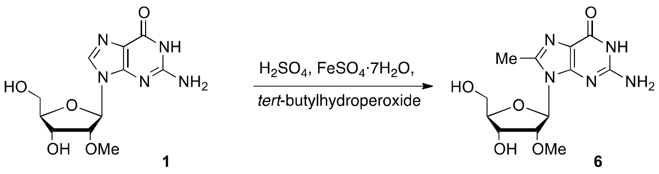

Scheme S2. Synthetic scheme of 2′-*O*-methyl-8-methylguanosine **6.**

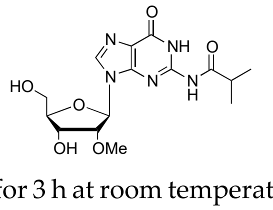

***N*²-Isobutyryl-2′-*O*-methylguanosine (2)** To a 2′-*O*-methylguanosine **1** (1 g, 3.4 mmol) dried three times by evaporation of pyridine (15 mL) and dissolved in dry pyridine (15 mL) was added trimethylchlorosilane (4.9 ml, 38.24 mmol). After the solution was stirred 30 minutes, isobutyric anhydride (6.34 mL, 38.24 mmol) was added, and the mixture was stirred for 3 h at room temperature. The reaction was cooled in an ice bath, and water (15 mL) was added. After 15 min, 29% aqueous ammonia (15 mL) was added, and the reaction was stirred for 15 min. The solution was then evaporated *in vacuo* and methanol (100 mL) was added to the residue. The precipitate (product) was filtered and dried, and the filtrate was concentrated in vacuo. The residue from the filtrate was purified by silica gel column chromatography (CH_2Cl_2 : MeOH = 10 : 1) to give the compound **3** (840 mg, 67%) as a solid. ^{1}H-NMR (400 MHz, D_2O) δ 8.21 (s, 1H), 6.08 (d, *J* = 4.9 Hz, 1H), 4.58 (t, *J* = 5.0 Hz, 1H), 4.42 (t, *J* = 5.0 Hz, 1H) , 4.19 (q, *J* = 3.3 Hz, 1H), 3.90 (dd, *J* = 3.2, 12.7 Hz, 1H), 3.82 (dd, *J* = 4.5, 12.7 Hz, 1H), 3.48 (s, 3H), 2.78 (sept, *J* = 6.9 Hz, 1H), 1.22 (d, *J* = 6.9 Hz, 3H), 1.22 (d, *J* = 6.9 Hz, 3H). HRMS (ESI) for $C_{15}H_{22}O_6N_5$ $[M+H]^+$: Calcd. 368.1565; Found. 368.1552.

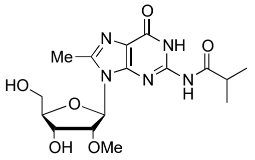

***N*²-Isobutyryl-2′-*O*-methyl-8-methylguanosine (3)** To a solution of compound **2** (1 g, 2.6 mmol) and of $FeSO_4·7H_2O$ (6.7 g, 24.1 mmol) in 160 mL of 1 N H_2SO_4 was added dropwise an aqueous solution (100 mL) containing 2.6 mL of 70% *tert*-butyl hydroperoxide (9.5 mmol) over a period of 5 min. After being stirred at 0 °C for 2 h, the reaction mixture was neutralized with saturated KOH solution. The supernatant obtained by centrifugation resulting in a brownish solid was triturated three times with 100 mL of methanol. The combined methanol solution was concentrated, and the residue was purified by silica gel column chromatography (CH_2Cl_2:MeOH = 10:1) to give the compound **3** (460 mg, 52%) as a solid. ^{1}H-NMR (400 MHz, D_2O) δ 5.96 (d, *J* = 5.4 Hz, 1H), 4.69 (t, *J* = 5.6 Hz, 1H), 4.11 (m, 1H), 3.90 (dd, *J* = 3.4, 12.6 Hz, 1H), 3.82 (dd, *J* = 5.1, 12.6 Hz, 1H), 3.43 (s, 3H), 2.78 (sept, *J* = 6.9 Hz, 1H), 2.57 (s, 3H), 1.21 (d, *J* = 6.9 Hz, 3H), 1.20 (d, *J* = 6.9 Hz, 3H). HRMS (ESI) for $C_{16}H_{22}O_6N_5$ $[M-H]^-$: Calcd. 380.1565; Found. 380.1574.

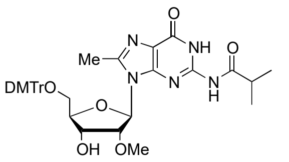

***N*²-Isobutyryl-5′-*O*-dimethoxytrityl-2′-*O*-methyl-8-methylguanosine (4)** To a compound **3** (487 mg, 1.26 mmol) dried three times by co-evaporation of pyridine (15 mL) and dissolved in dry pyridine (15 mL) was added 4,4′-dimethoxytritylchloride (600 mg, 1.77 mmol), triethylamine (246 μL, 1.77 mmol) and 4-(dimethylamino)pyridine (5 mg, 0.038 mmol). After 12 h, the solution was evaporated *in vacuo*, and the residue was dissolved in dichloromethane (50 ml) and added aqueous 5%-$NaHCO_3$ solution. The mixture was extracted three times with dichloromethane. The organic layer was dried over Na_2SO_4, and concentrated in vacuo. The residue was purified by silica gel column chromatography (*n*-hex:AcOEt = 1:2) to give the compound **4** (780 mg, 94%) as a solid. ^{1}H-NMR

(400 MHz, $CDCl_3$) δ 11.90 (s, 1H), 7.59-7.57 (m, 1H), 7.44-7.39 (m, 4H), 7.29-7.16 (m, 4H), 6.80-6.75 (m, 4H), 5.76 (d, *J* = 7.1 Hz, 1H), 5.20 (dd, *J* = 5.4, 7.0 Hz, 1H), 4.66 (m, 1H), 4.15 (m, 1H), 3.77 (s, 3H), 3.75 (s, 3H), 3.55 (dd, *J* = 1.7, 10.8 Hz, 1H), 3.48 (s, 3H), 3.03 (dd, *J* = 3.2, 10.8 Hz, 1H), 2.60 (s, 3H), 1.09 (sept, *J* = 6.8 Hz, 1H), 0.78 (d, *J* = 6.8 Hz, 3H), 0.45 (d, *J* = 6.8 Hz, 3H). HRMS (ESI) for $C_{37}H_{42}O_8N_5$ $[M+H]^+$: Calcd. 684.3028; Found. 684.3016.

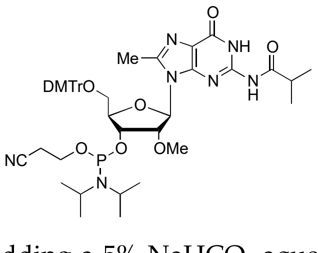

***N*²-Isobutyryl-5′-*O*-dimethoxytrityl-2′-*O*-methyl-8-methylguanosine phosphoramidite (5)** The compound **4** (780 mg, 1.14 mmol) was treated with dry *N,N*-diisopropylethylamine (794 μL, 4.56 mmol) and 2-cyanoethyl-*N,N*-diisopropylchlorophosphoramidite (500 μL, 2.28 mmol) in dry acetonitrile (10 mL) and stirred at room temperature for 2 h. After addition of dichloromethane (50 mL), the reaction was stopped by adding a 5% $NaHCO_3$ aqueous solution (50 mL). The aqueous layer was extracted three times with dichloromethane (100 mL). The combined organic layers were dried over Na_2SO_4 and the solvent was evaporated *in vacuo*. The residue was purified by silica gel column chromatography (CH_2Cl_2:AcOEt = 3:1) to give the compound **5** (700 mg, 67%) as a solid. ^{1}H-NMR (400 MHz, DMSO-d_6) δ 11.92 (s, 1H), 7.59-7.56 (m, 2H), 7.46-7.40 (m, 4H), 7.26-7.21 (m, 3H), 6.80-6.75 (m, 4H), 5.81 (d, *J* = 8.3 Hz, 2H), 5.73 (d, *J* = 6.9 Hz, $2H_{isomer}$), 5.15 (dd, *J* = 5.2, 8.0 Hz, 1H), 5.00 (m, $1H_{isomer}$), 4.70-4.64 (m, 1H), 4.27 (m, 1H), 4.18 (m, $1H_{isomer}$), 4.02-3.88 (m, 1H), 3.77 (s, 3H), 3.75 (s, 3H), 3.61-3.51 (m, 6H), 3.46 (s, 3H), 3.43 (s, $3H_{isomer}$), 3.10 (dd, *J* = 4.3, 10.7 Hz, 1H), 3.02 (dd, *J* = 3.4, 10.7 Hz, $1H_{isomer}$), 2.75-2.63 (m, 1H), 1.21 (d, *J* = 6.8 Hz, $8H_{isomer}$), 1.16 (d, *J* = 6.8 Hz, 8H), 0.96 (d, *J* = 6.8 Hz, 4H), 0.81 (d, *J* = 6.8 Hz, $3H_{isomer}$), 0.80 (d, *J* = 6.8 Hz, 3H), 0.55 (d, *J* = 6.8 Hz, $3H_{isomer}$), 0.5 (d, *J* = 6.8 Hz, 3H). ^{31}P-NMR (161 MHz, $CDCl_3$) δ 150.26, 150.16. HRMS (ESI) for $C_{46}H_{57}O_9N_7P$ $[M-H]^-$: Calcd. 882.3950; Found. 882.3969.

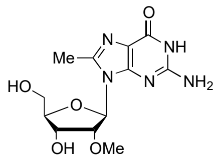

2′-*O*-methyl-8-methylguanosine (6) To a solution of 2′-*O*-methylguanosine **1** (1 g, 2.6 mmol) and of $FeSO_4 \cdot 7H_2O$ (6.7 g, 24.1 mmol) in 160 mL of 1 N H_2SO_4 was added dropwise an aqueous solution (100 mL) containing 2.6 mL of 70% *tert*-butyl hydroperoxide (9.5 mmol) over a period of 5 min. After being stirred at 0 °C for 2 h, the reaction mixture was neutralized with saturated KOH solution. The supernatant obtained by centrifugation resulting in a brownish solid was triturated three times with 100 mL of methanol. The combined methanol solution was concentrated, and the residue was purified by silica gel column chromatography (CH_2Cl_2 : MeOH = 10 : 1) to give the compound **3** (500 mg, 48%) as a solid. ^{1}H-NMR (400 MHz, CD_3OD) δ 5.84 (d, *J* = 6.9 Hz, 1H), 4.64-4.61 (m, 1H), 4.49 (dd, *J* = 2.5, 5.2 Hz, 1H), 4.11 (m, 1H), 3.86 (dd, *J* = 2.9, 12.4 Hz, 1H), 3.73 (dd, *J* = 3.1, 12.4 Hz, 1H), 3.40 (s, 3H), 2.47 (s, 3H). HRMS (ESI) for $C_{12}H_{16}N_5O_5$ [M–H]–: Calcd. 310.1146; Found. 310.1153.

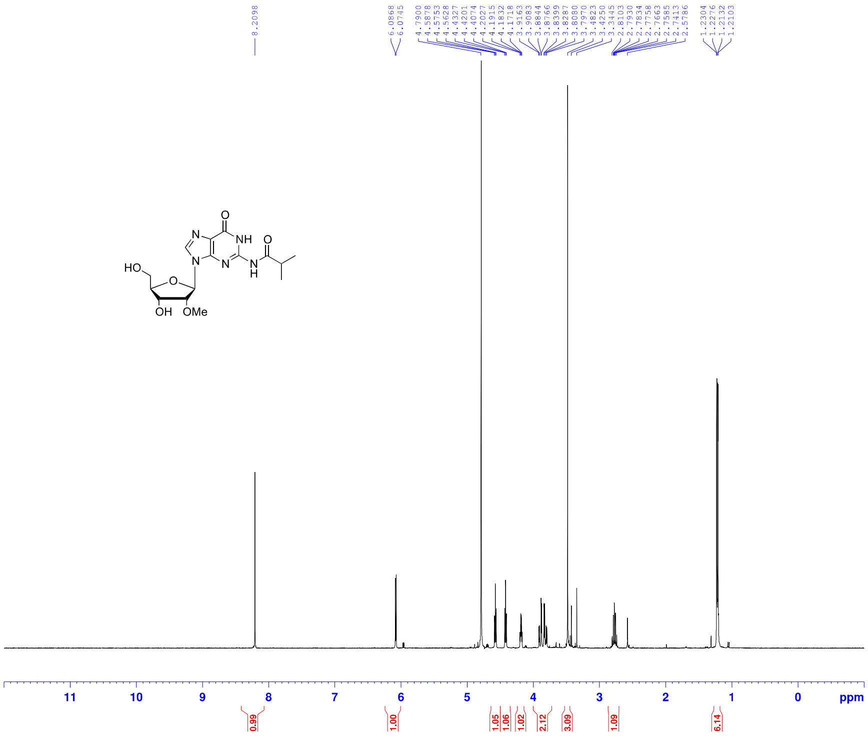

Figure S1. ^{1}H NMR spectrum of compound **2**.

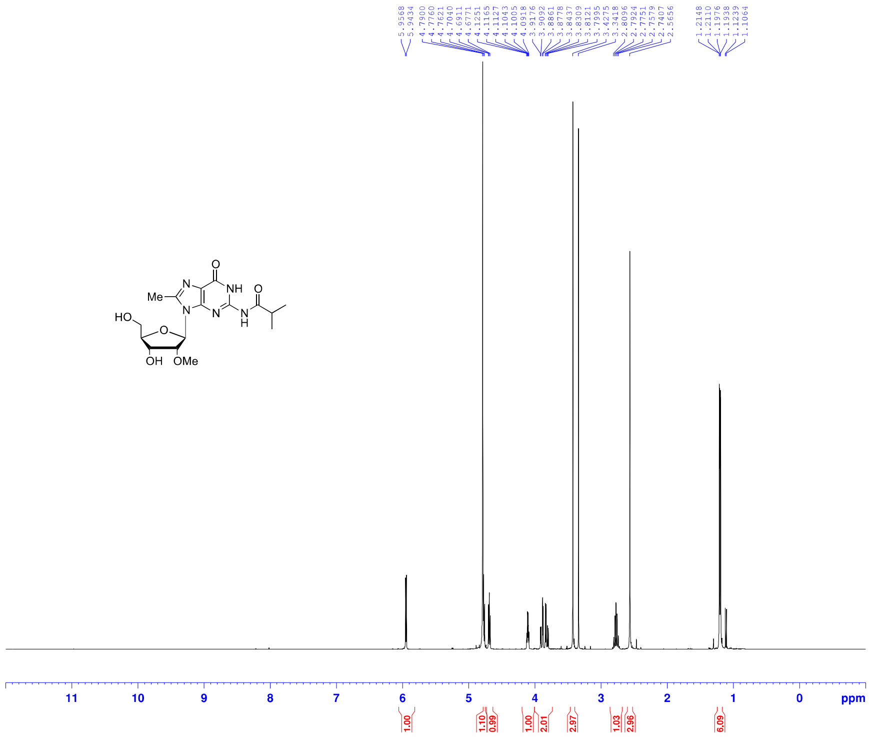

Figure S2. ^{1}H NMR spectrum of compound **3**.

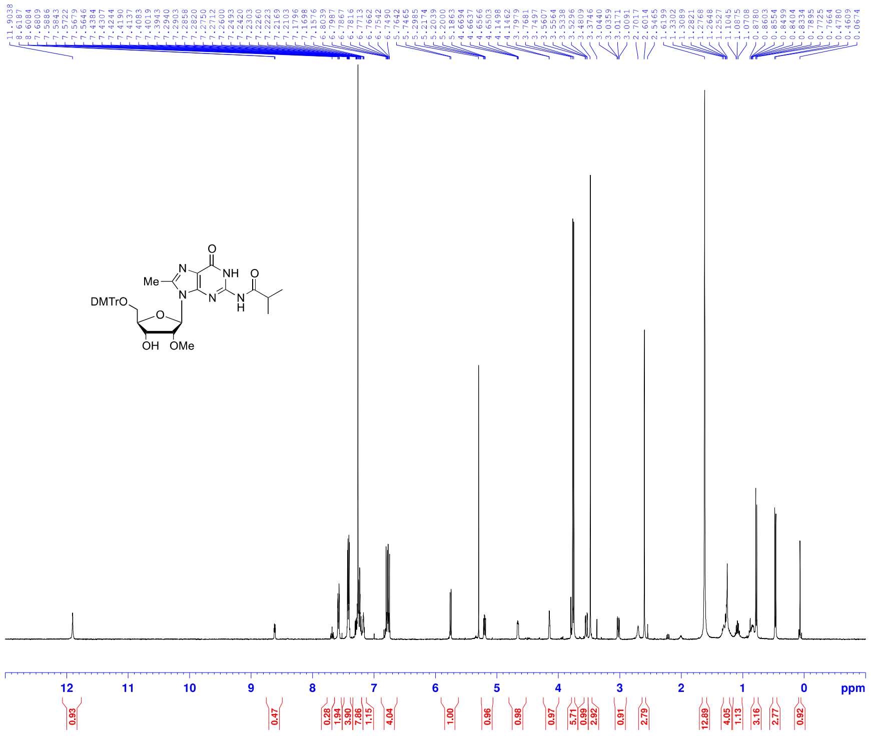

Figure S3. ^{1}H NMR spectrum of compound **4**.

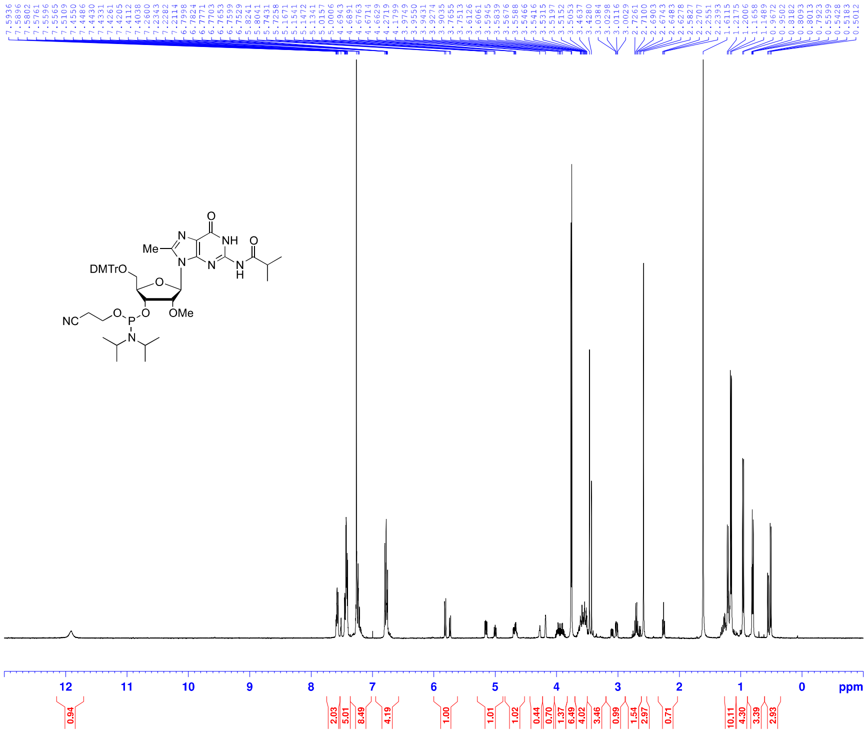

Figure S4. ^{1}H NMR spectrum of compound **5**.

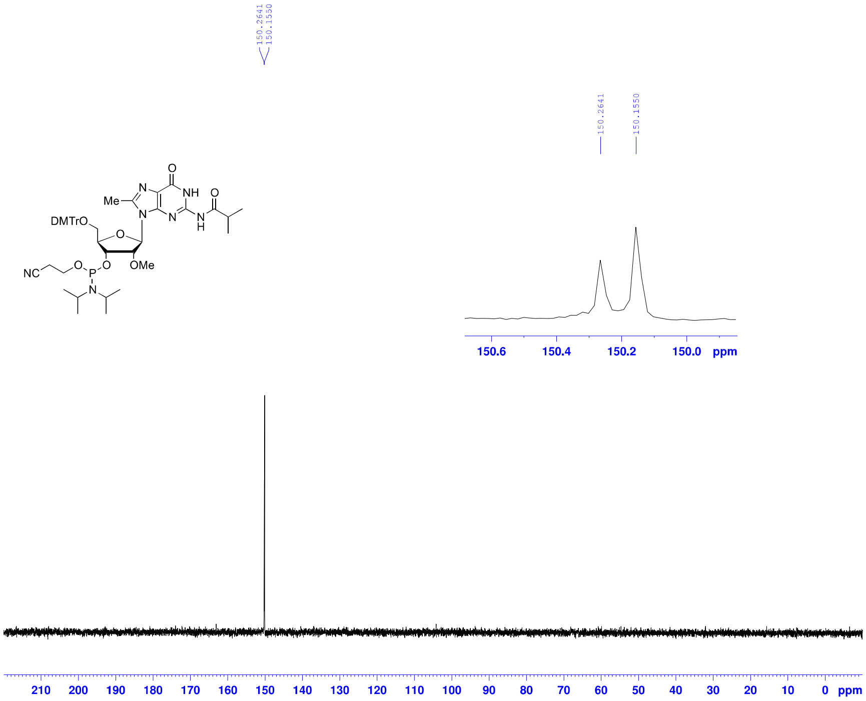

Figure S5. ^{31}P NMR spectrum of compound **5**.

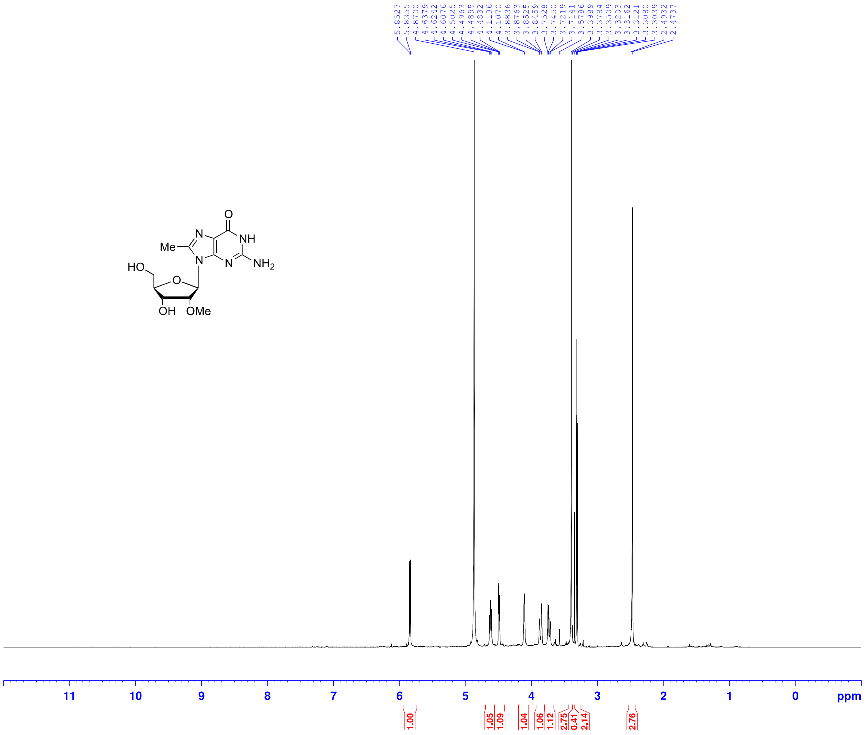

Figure S6. ^{1}H NMR spectrum of compound **6**.

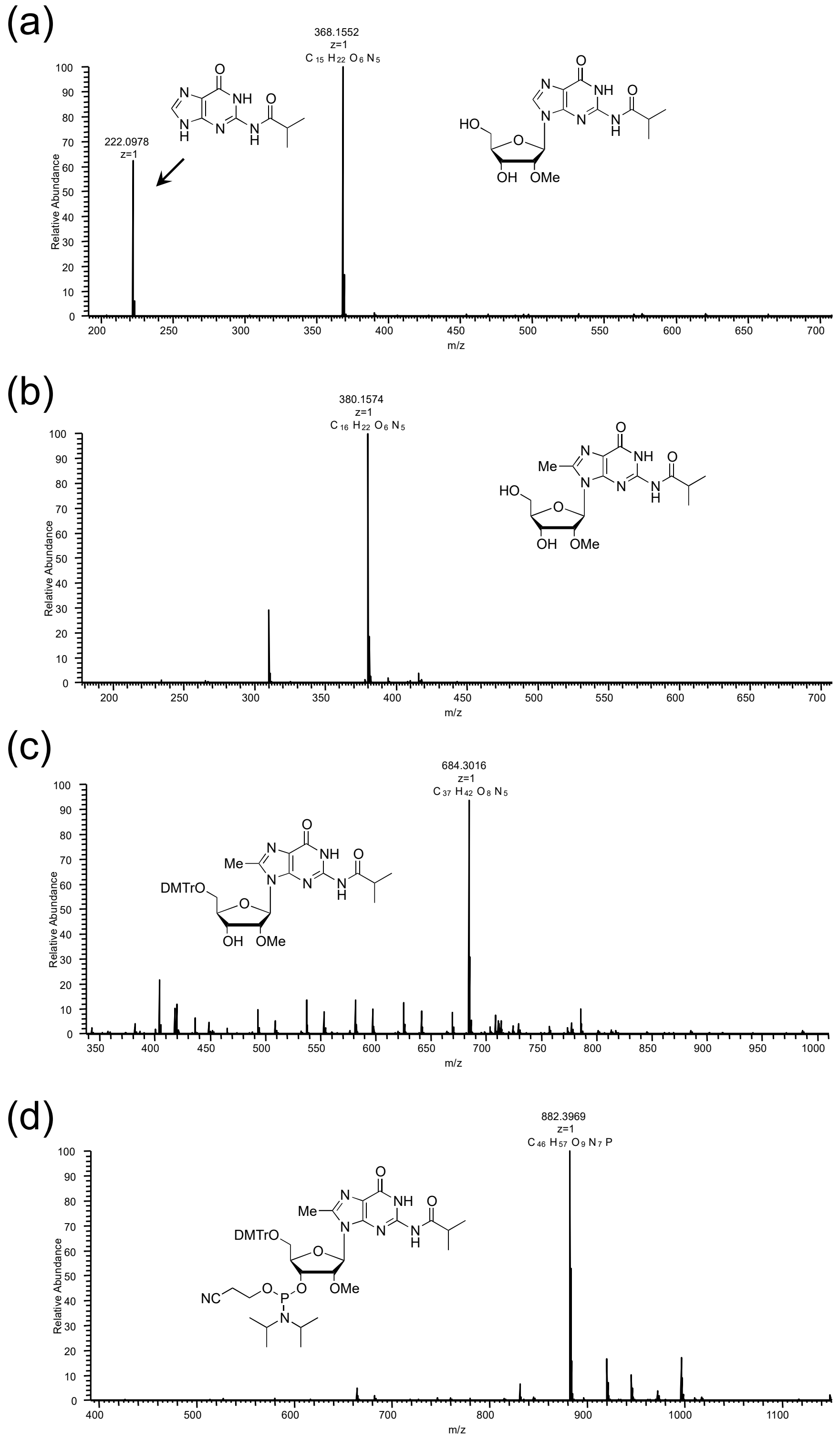

Figure S7. HRMS spectra of compound **2** (**a**), **3** (**b**), **4** (**c**), **5** (**d**).

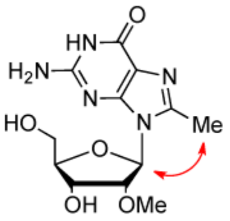

m[8m]G

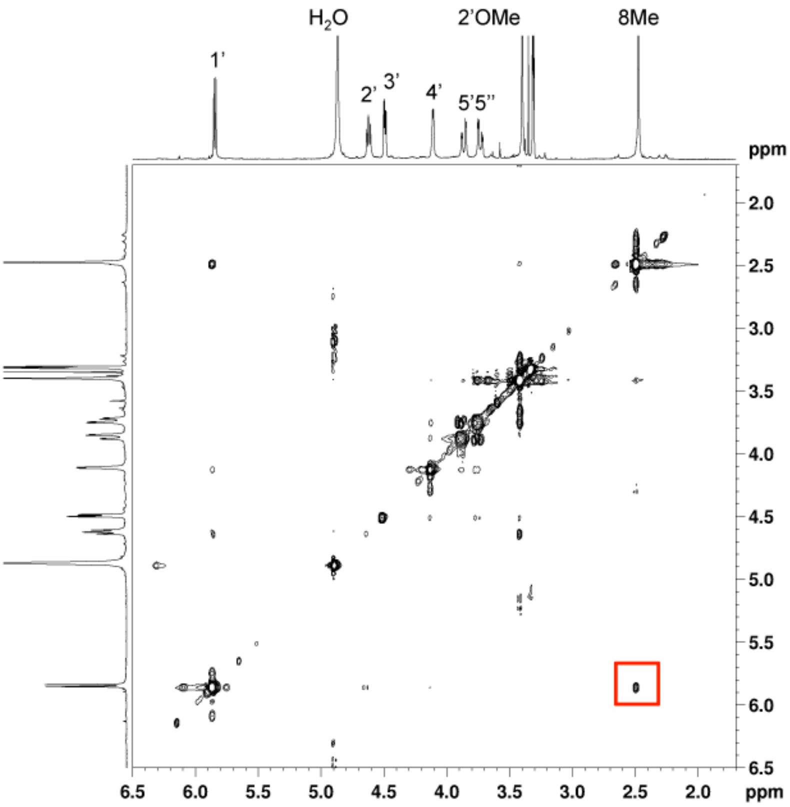

Figure S8. *Syn* structure and NOESY spectrum of 2′-*O*-methyl-8-methyl guanosine (m[8]Gm), the red square indicated the nuclear Overhauser effect (NOE) between C1'H and $8CH_3$. NOE measurement ([m[8]Gm] = 5 mM) was performed in D_2O at 20 °C.

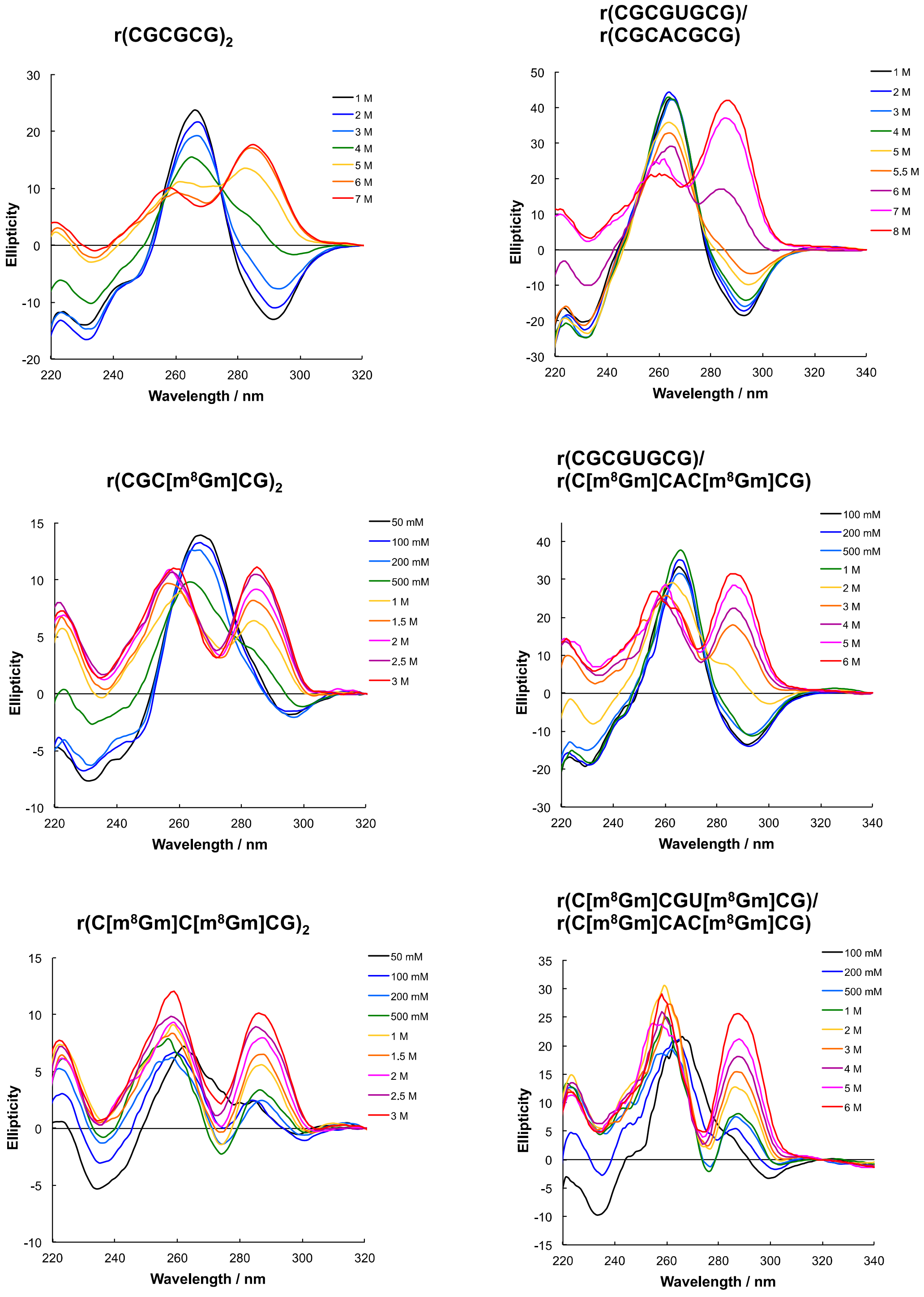

Figure S9. CD spectra of RNA shown in Table 1 in 5 mM sodium phosphate buffer (pH 7.0) at 10 °C at various $NaClO_4$ concentrations.

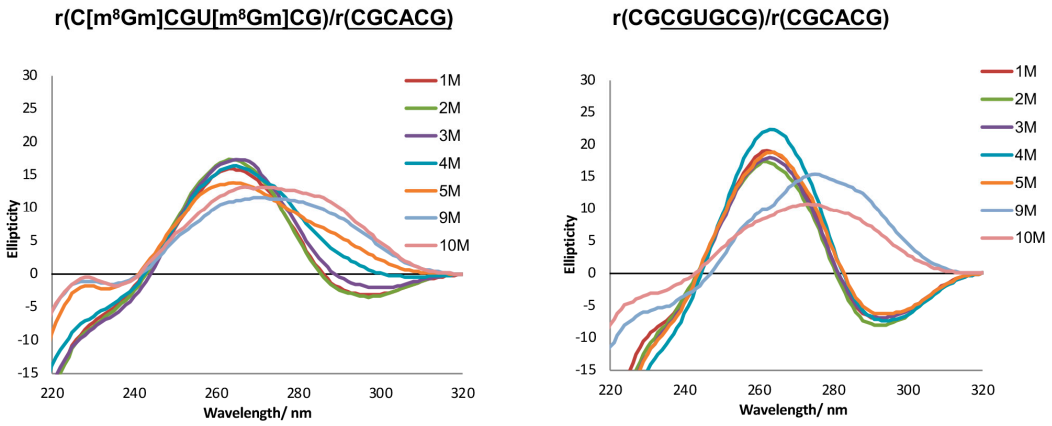

Figure S10. CD spectra of r(C[m^{8}Gm]CGU[m^{8}Gm]CG)/r(CGCACG) (a) and r(CGCGUGCG)/r(CGCACG) (b) at 10 °C with various $NaClO_4$ concentrations in 5 mM sodium phosphate buffer (pH 7.0). The midpoint for RNA containing one m^{8}Gm was 4000 mM, the native RNA was higher than 6000 mM.

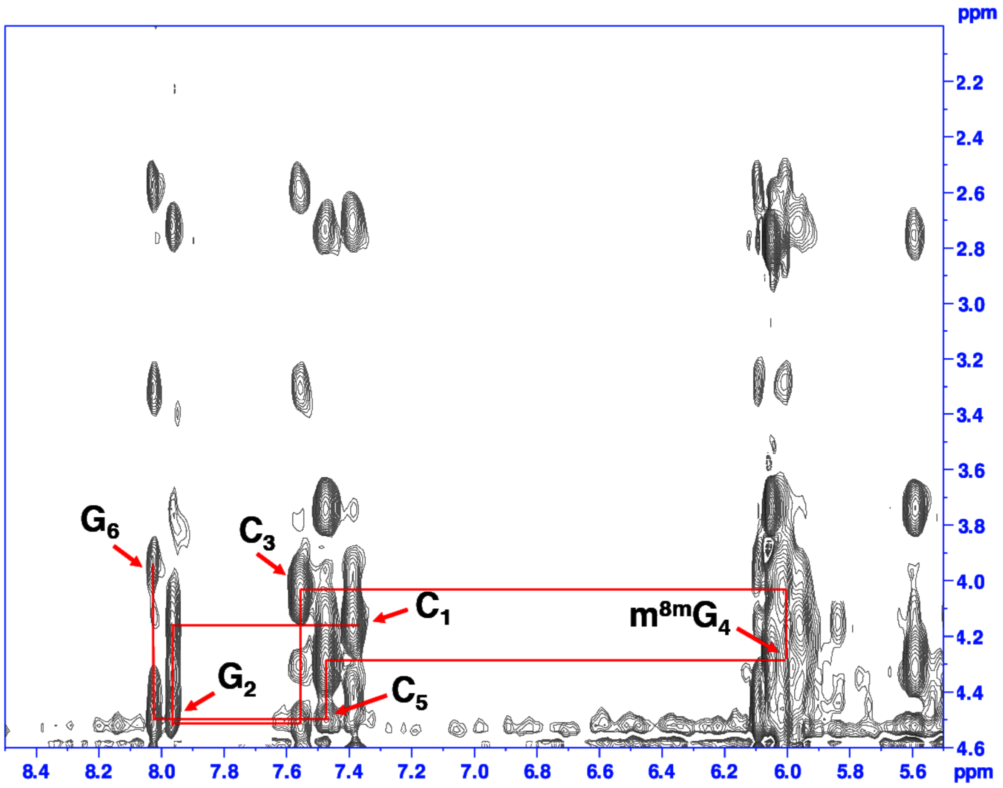

Figure S11. H6/H2′ of C and H8/H2′ of G (8CH_3/H2′ of m^{8}Gm) proton region of NOESY spectra of r(CGC[m^{8}Gm]CG)$_2$ in $NaClO_4$ solution. The NOE connectivity pathway is shown as red line. Intraresidue NOE cross-peaks are labeled with residue numbers.

Table S1. ^{1}H NMR chemical shifts δ_H (p.p.m.) of r(CGC[m^{8}Gm]CG)$_2$ Z-RNA.

Residue	H8(G) H6(C)	CH_3(G$_4$) H5(C)	H1′	H2′	H3′	H4′	H5′	H5′′	OCH_3	imino	amino	
C$_1$	7.38	5.27	5.99	4.14	4.39	3.93	3.88	2.73	–	–	–	6.33
G$_2$	7.97	–	6.02	4.51	5.33	4.40	3.92	3.88	–	13.13	8.75	–
C$_3$	7.55	5.44	6.01	4.03	4.68	3.92	3.34	2.58	–	–	–	6.28
m^{8}Gm$_4$	–	2.75	6.05	4.27	5.44	4.33	3.92	3.89	3.73	13.11	8.75	–
C$_5$	7.48	5.60	6.03	4.45	4.50	3.94	3.95	2.75	–	–	–	6.44
G$_6$	8.03	–	6.09	3.96	4.84	4.32	3.92	3.88	–	13.34	8.74	–
